# Supplementary material for: Process Evaluation of the ‘No Money No Time’ Healthy Eating Website Promoted Using Social Marketing Principles. A Case Study
Source: Int J Environ Res Public Health. 2021 Mar 30;18(7):3589. doi: 10.3390/ijerph18073589 (PMC8038032; doi:10.3390/ijerph18073589)
Supplement: Supplementary file 1 [file ijerph-18-03589-s001.pdf]

International Journal of
Environmental Research and Public Health

MDPI

Supplementary Materials

Contents	Page
Figure S1: Section from No Money No Time Homepage	3
Figure S2: Section of dietary feedback report from the embedded dietary assessment tool—Healthy Eating Quiz	3
Figure S3: Section of recipe page on No Money No Time with filters	4
Figure S4: Section of personal dashboard on No Money No Time	4
Figure S5: Engagement information from Google Analytics for all users of No Money No Time.	5
Figure S6: Funnel report to map out the customer journey on No Money No Time website from 17 July 2019 to 17 July 2020	6
Figure S7: Engagement information from Google Analytics for return users vs new users of No Money No Time.	7
Figure S8: Engagement information from Google Analytics for young adults 18–34 years compared to the rest of sample from No Money No Time.	8
Figure S9: User-acquisition data from Google Analytics by channel for new users and returning users.	9
Figure S10: User-acquisition and engagement data from Google Analytics by channel for young adults (18–34 years) and all other users.	10
Figure S11: User-acquisition and engagement data from Google Analytics by device type for young adults (18–34 years) and all other users.	11
Figure S12: Mobile device use for all users accessing No Money No Time from 17 July 2019 to 17 July 2020	12
Figure S13: Mobile device use for young adults (18–34 years) accessing No Money No Time from 17 July 2019 to 17 July 2020	13
Figure S14: Google analytics 'benchmarking' comparison of traffic acquisition for 'No Money No Time' with aggregated industry data from 13,034 food and drink websites from 17 July 2019 to 17 July 2020	14
Figure S15: Google analytics 'benchmarking' comparison of traffic acquisition 'No Money No Time' with aggregated industry data from 1830 websites with 'Cooking & Recipes' category from 17 July 2019 to 17 July 2020	15
Figure S16: Google analytics 'benchmarking' comparison of device type recruitment and engagement data of 'No Money No Time' with aggregated industry data from 13,034 food and drink websites from 17 July 2019 to 17 July 2020	16

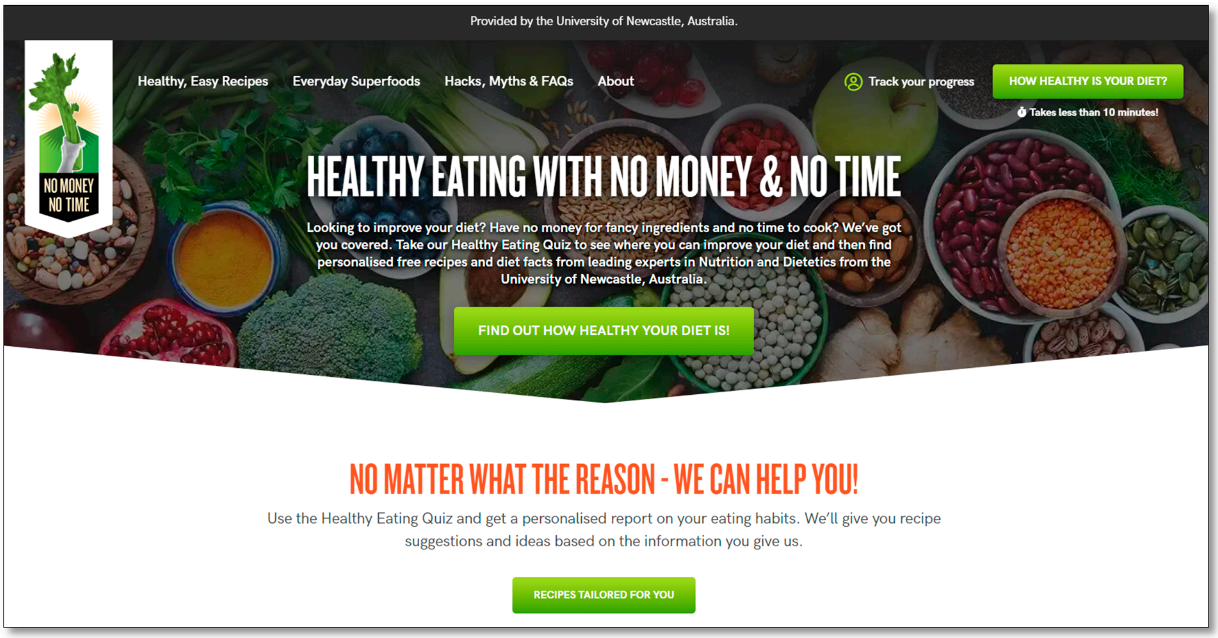

Figure 1. Section from No Money No Time Homepage.

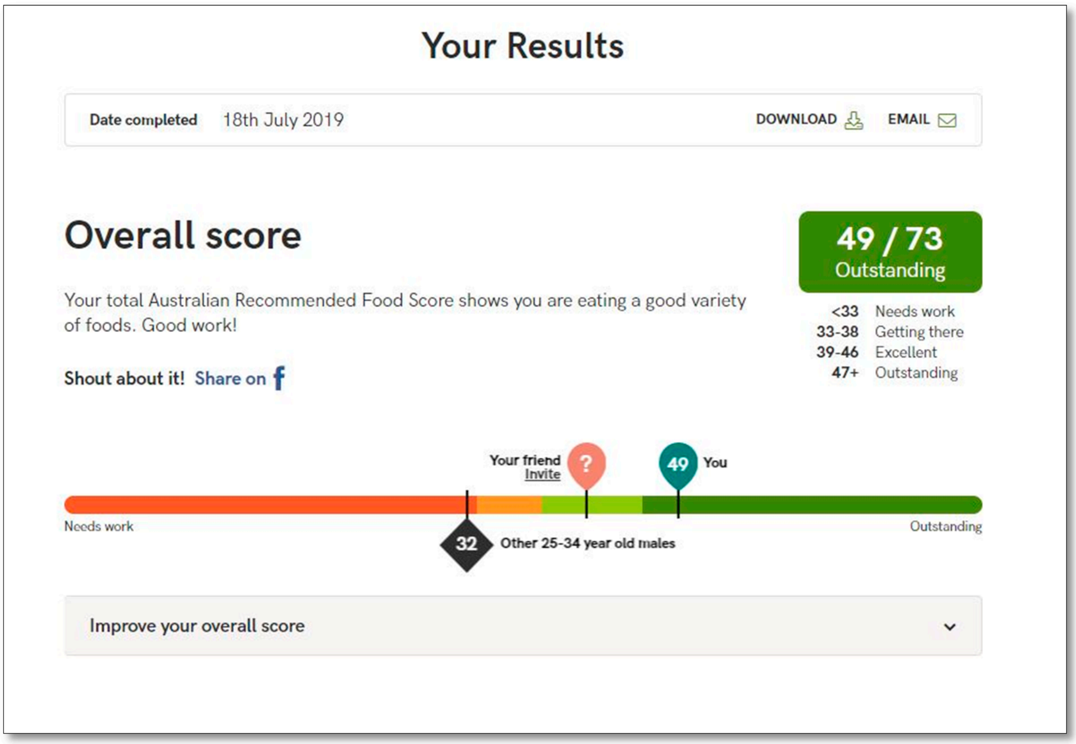

Figure 2. Section of dietary feedback report from the embedded dietary assessment tool—Healthy Eating Quiz.

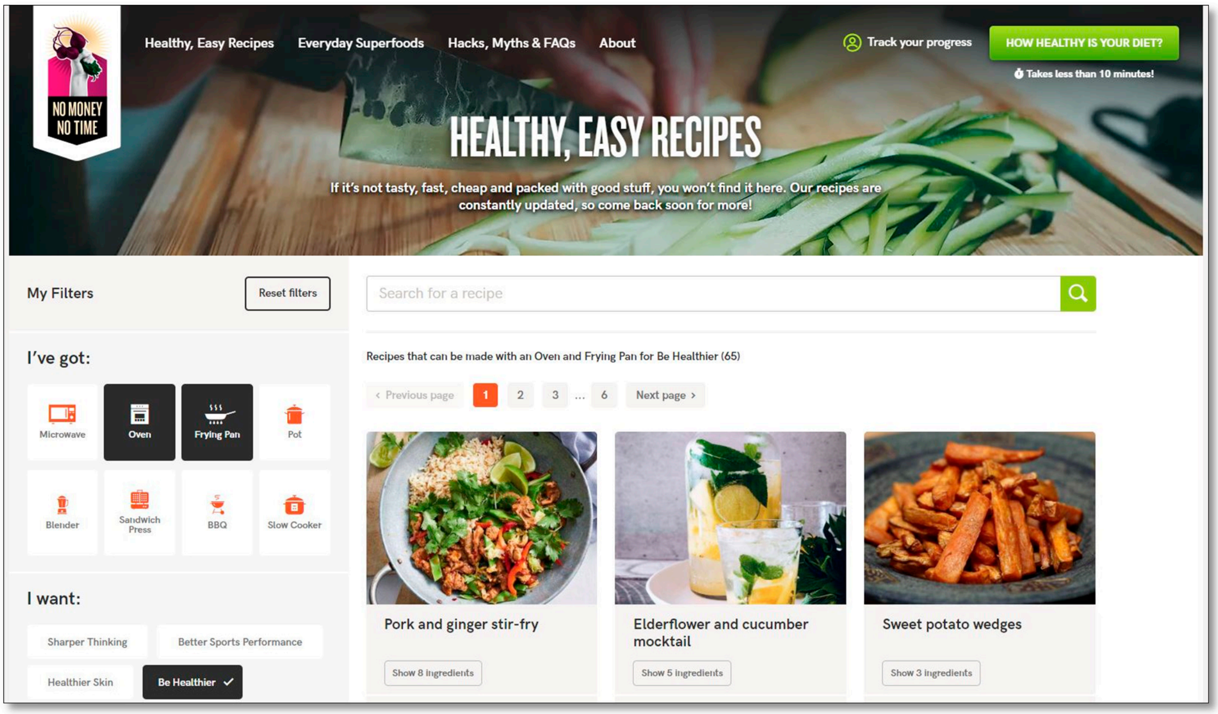

Figure 3. Section of recipe page on No Money No Time with filters.

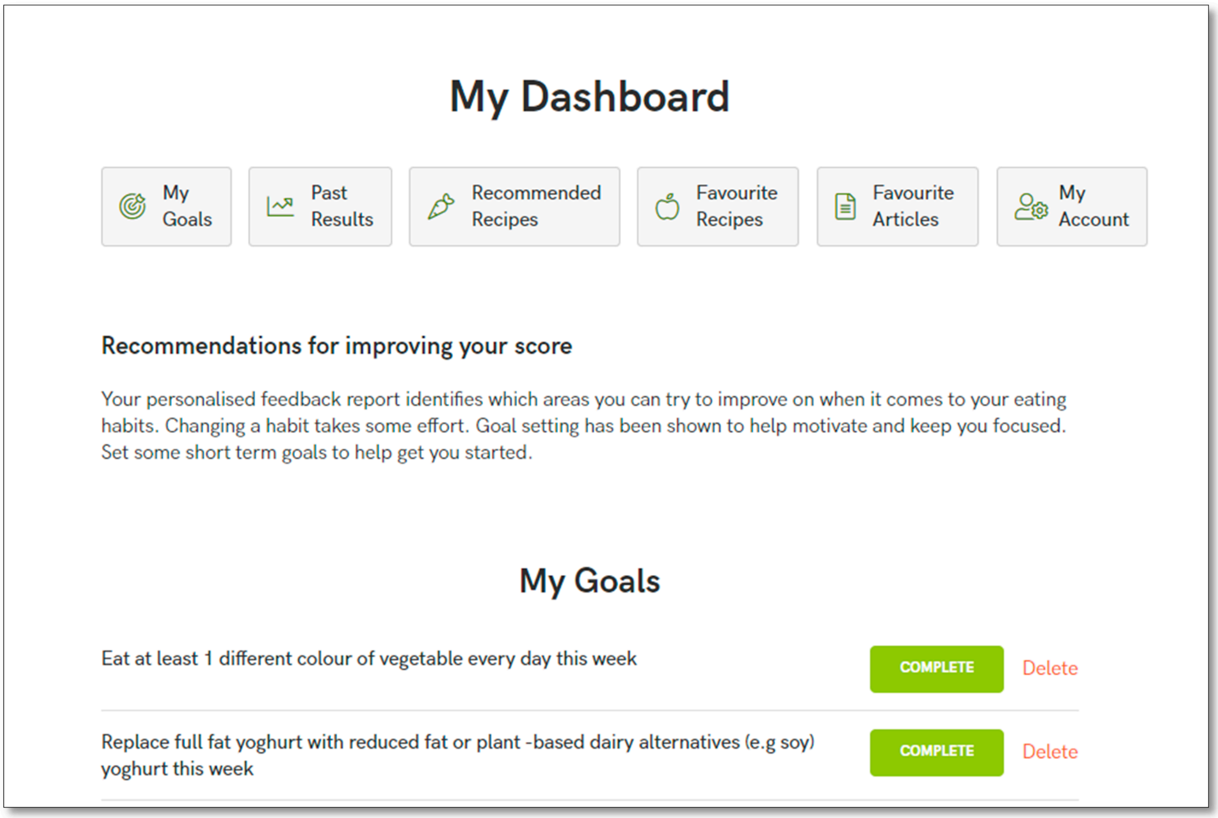

Figure 4. Section of personal dashboard on No Money No Time.

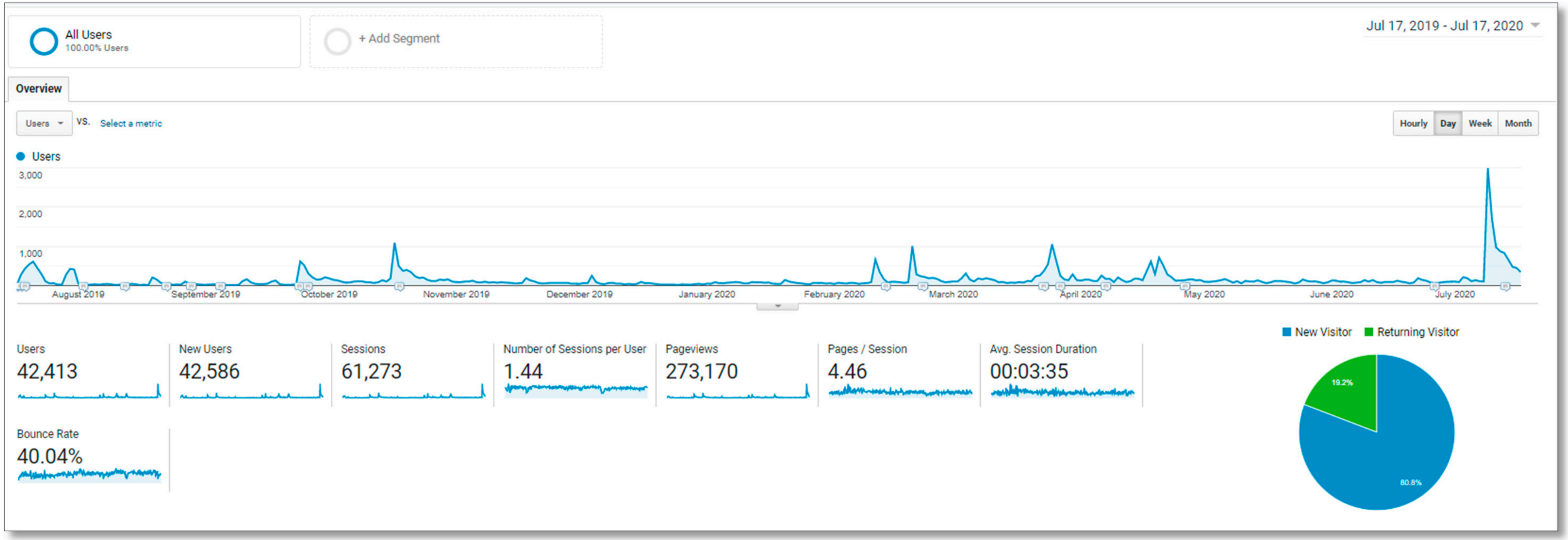

Figure 5. Engagement information from Google Analytics for all users of No Money No Time.

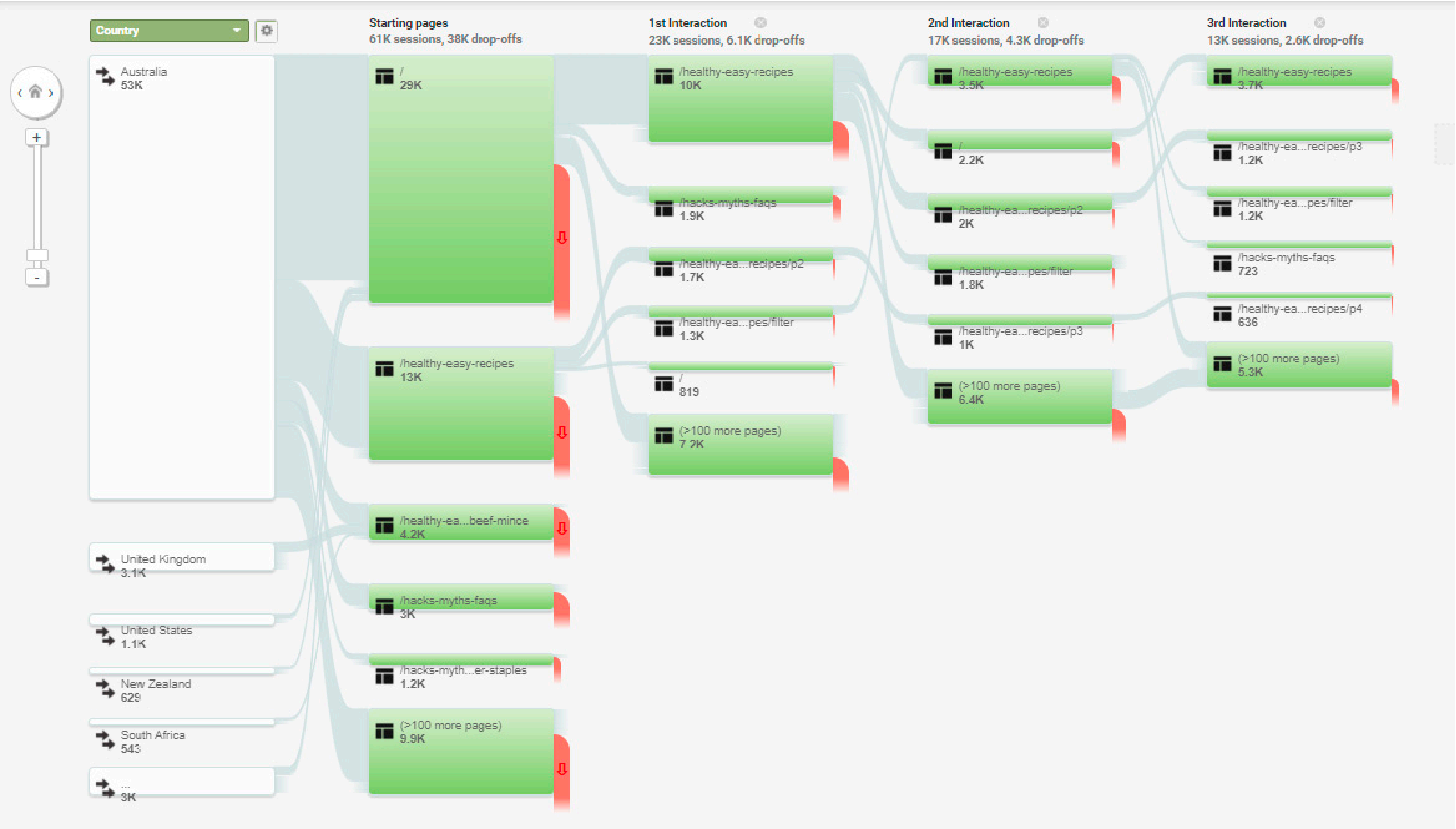

Figure 6. Funnel report to map out the customer journey on No Money No Time website from 17 July 2019 to 17 July 2020.

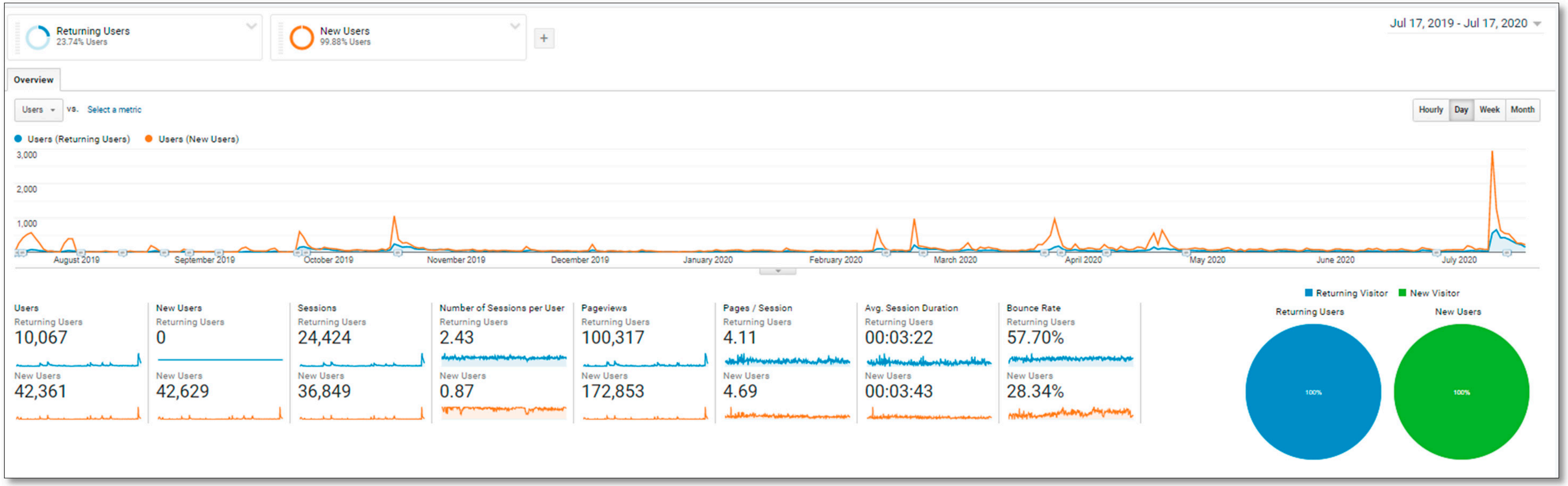

Figure 7. Engagement information from Google Analytics for return users vs new users of No Money No Time.

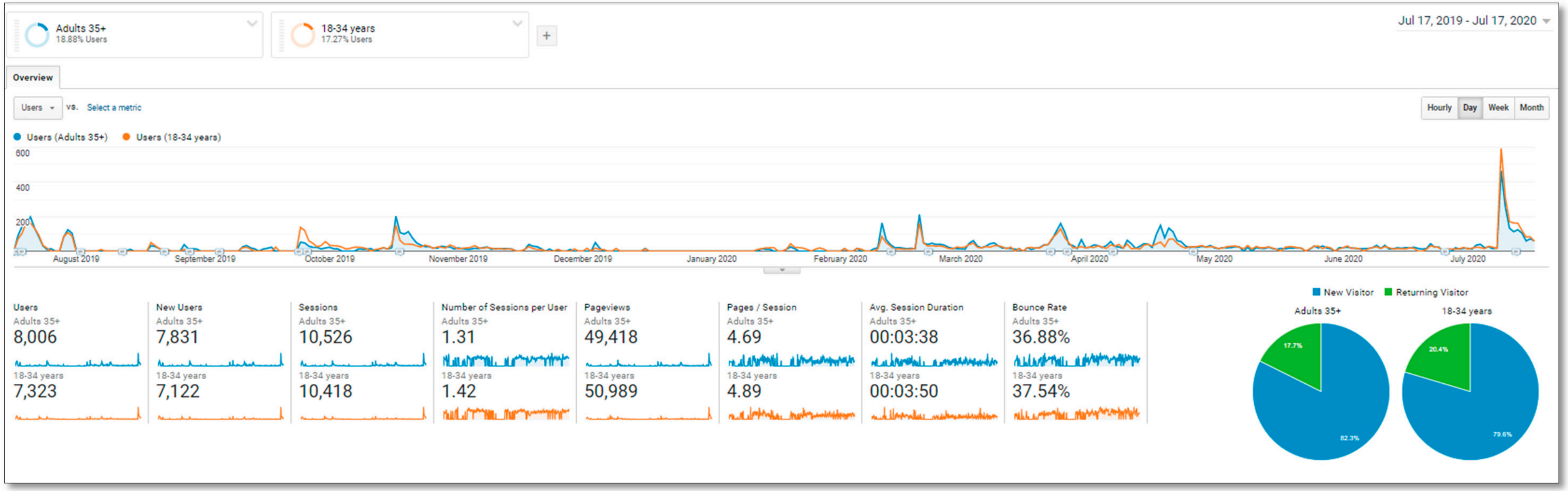

Figure 8. Engagement information from Google Analytics for young adults 18–34 years compared to the rest of sample from No Money No Time.

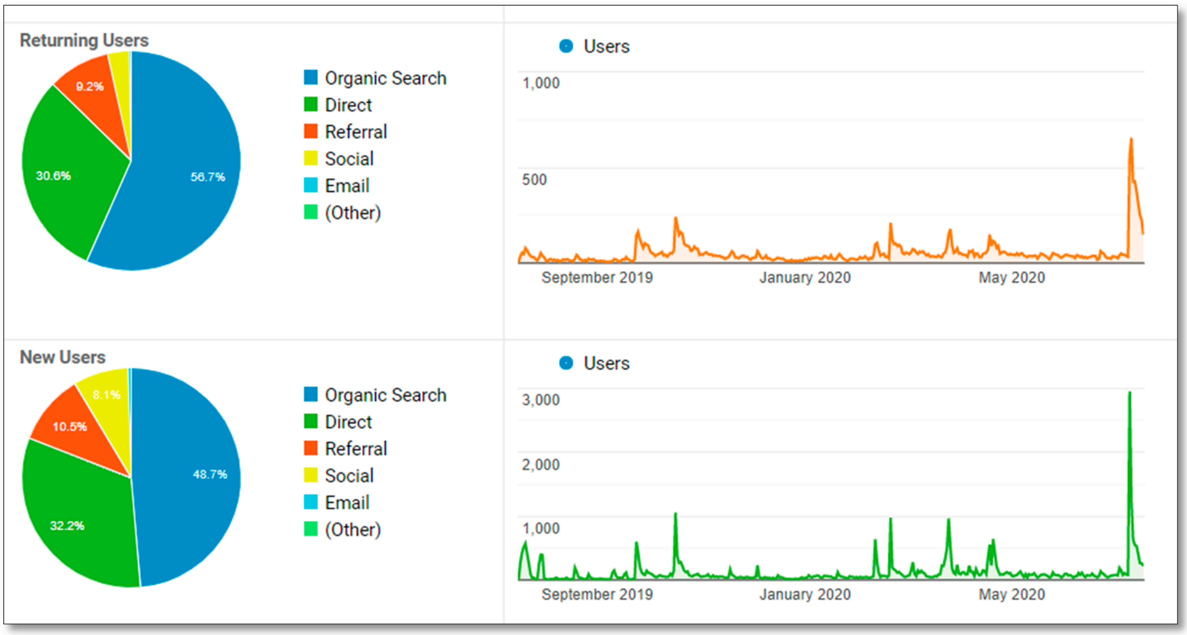

Figure 9. User-acquisition data from Google Analytics by channel for new users and returning users.

Default Channel Grouping	Acquisition			Behavior			Conversions Goal 1: Clicks Quiz Call to Action
	Users	New Users	Sessions	Bounce Rate	Pages / Session	Avg. Session Duration	Clicks Quiz Call to Action (Goal 1 Conversion Rate)
**Adults 35+**	8,006 % of Total: 18.88% (42,413)	7,826 % of Total: 18.36% (42,629)	10,526 % of Total: 17.18% (61,273)	36.88% Avg for View: 40.04% (-7.90%)	4.69 Avg for View: 4.46 (5.31%)	00:03:38 Avg for View: 00:03:35 (1.34%)	24.20% Avg for View: 25.10% (-3.59%)
**18-34 years**	7,323 % of Total: 17.27% (42,413)	7,122 % of Total: 16.71% (42,629)	10,418 % of Total: 17.00% (61,273)	37.54% Avg for View: 40.04% (-6.25%)	4.89 Avg for View: 4.46 (9.78%)	00:03:50 Avg for View: 00:03:35 (6.99%)	26.20% Avg for View: 25.10% (4.40%)
1. Organic Search							
Adults 35+	**3,564** (43.38%)	3,362 (42.96%)	5,185 (49.26%)	38.55%	5.07	00:03:56	26.11%
18-34 years	**3,778** (50.49%)	3,586 (50.35%)	6,117 (58.72%)	38.68%	5.22	00:04:04	27.17%
2. Direct							
Adults 35+	**1,890** (23.01%)	1,834 (23.43%)	2,539 (24.12%)	34.50%	4.59	00:03:34	22.25%
18-34 years	**1,614** (21.57%)	1,566 (21.99%)	2,318 (22.25%)	36.28%	4.61	00:03:38	24.20%
3. Referral							
Adults 35+	**1,653** (20.12%)	1,589 (20.30%)	1,747 (16.60%)	36.41%	4.60	00:03:38	17.52%
18-34 years	**1,382** (18.47%)	1,303 (18.30%)	1,212 (11.63%)	37.79%	4.73	00:03:46	18.73%
4. Social							
Adults 35+	**1,060** (12.90%)	996 (12.73%)	1,000 (9.50%)	34.60%	3.32	00:02:19	31.90%
18-34 years	**696** (9.30%)	656 (9.21%)	758 (7.28%)	31.66%	3.41	00:02:44	36.81%
5. Email							
Adults 35+	**48** (0.58%)	45 (0.58%)	55 (0.52%)	45.45%	2.47	00:01:13	5.45%
18-34 years	**13** (0.17%)	11 (0.15%)	13 (0.12%)	46.15%	2.62	00:00:23	7.69%

Figure 10. User-acquisition and engagement data from Google Analytics by channel for young adults (18–34 years) and all other users.

Device Category	Acquisition		
	Users	New Users	Sessions
**Adults 35+**	8,006<br>% of Total: 18.88% (42,413)	7,831<br>% of Total: 18.37% (42,629)	10,526<br>% of Total: 17.18% (61,273)
**18-34 years**	7,323<br>% of Total: 17.27% (42,413)	7,122<br>% of Total: 16.71% (42,629)	10,418<br>% of Total: 17.00% (61,273)
1. mobile			
Adults 35+	**4,324** (53.96%)	4,251 (54.28%)	5,551 (52.74%)
18-34 years	**4,357** (59.62%)	4,265 (59.88%)	6,457 (61.98%)
2. desktop			
Adults 35+	**2,903** (36.23%)	2,823 (36.05%)	3,845 (36.53%)
18-34 years	**2,837** (38.82%)	2,748 (38.58%)	3,762 (36.11%)
3. tablet			
Adults 35+	**786** (9.81%)	757 (9.67%)	1,130 (10.74%)
18-34 years	**114** (1.56%)	109 (1.53%)	199 (1.91%)

Figure 11. User-acquisition and engagement data from Google Analytics by device type for young adults (18–34 years) and all other users.

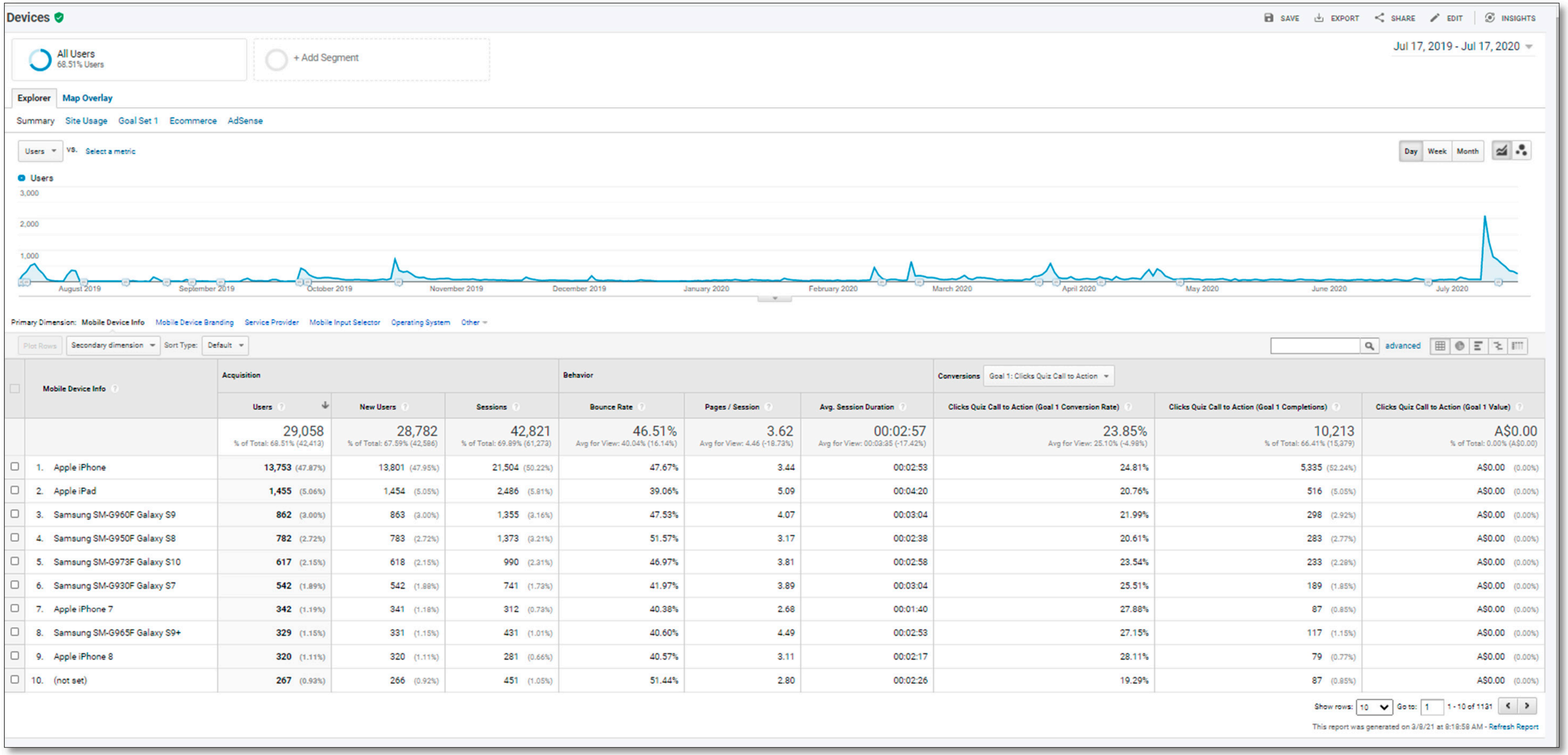

Mobile Device Info	Acquisition: Users	Acquisition: New Users	Acquisition: Sessions	Behavior: Bounce Rate	Behavior: Pages / Session	Behavior: Avg. Session Duration	Conversions: Clicks Quiz Call to Action (Goal 1 Conversion Rate)	Conversions: Clicks Quiz Call to Action (Goal 1 Completions)	Conversions: Clicks Quiz Call to Action (Goal 1 Value)
	29,058 % of Total: 68.51% (42,413)	28,782 % of Total: 67.59% (42,586)	42,821 % of Total: 69.89% (61,273)	46.51% Avg for View: 40.04% (16.14%)	3.62 Avg for View: 4.46 (-18.73%)	00:02:57 Avg for View: 00:03:35 (-17.42%)	23.85% Avg for View: 25.10% (-4.98%)	10,213 % of Total: 66.41% (15,379)	A$0.00 % of Total: 0.00% (A$0.00)
1. Apple iPhone	13,753 (47.87%)	13,801 (47.95%)	21,504 (50.22%)	47.67%	3.44	00:02:53	24.81%	5,335 (52.24%)	A$0.00 (0.00%)
2. Apple iPad	1,455 (5.06%)	1,454 (5.05%)	2,486 (5.81%)	39.06%	5.09	00:04:20	20.76%	516 (5.05%)	A$0.00 (0.00%)
3. Samsung SM-G960F Galaxy S9	862 (3.00%)	863 (3.00%)	1,355 (3.16%)	47.53%	4.07	00:03:04	21.99%	298 (2.92%)	A$0.00 (0.00%)
4. Samsung SM-G950F Galaxy S8	782 (2.72%)	783 (2.72%)	1,373 (3.21%)	51.57%	3.17	00:02:38	20.61%	283 (2.77%)	A$0.00 (0.00%)
5. Samsung SM-G973F Galaxy S10	617 (2.15%)	618 (2.15%)	990 (2.31%)	46.97%	3.81	00:02:58	23.54%	233 (2.28%)	A$0.00 (0.00%)
6. Samsung SM-G930F Galaxy S7	542 (1.89%)	542 (1.88%)	741 (1.73%)	41.97%	3.89	00:03:04	25.51%	189 (1.85%)	A$0.00 (0.00%)
7. Apple iPhone 7	342 (1.19%)	341 (1.18%)	312 (0.73%)	40.38%	2.68	00:01:40	27.88%	87 (0.85%)	A$0.00 (0.00%)
8. Samsung SM-G965F Galaxy S9+	329 (1.15%)	331 (1.15%)	431 (1.01%)	40.60%	4.49	00:02:53	27.15%	117 (1.15%)	A$0.00 (0.00%)
9. Apple iPhone 8	320 (1.11%)	320 (1.11%)	281 (0.66%)	40.57%	3.11	00:02:17	28.11%	79 (0.77%)	A$0.00 (0.00%)
10. (not set)	267 (0.93%)	266 (0.92%)	451 (1.05%)	51.44%	2.80	00:02:26	19.29%	87 (0.85%)	A$0.00 (0.00%)

Show rows: 10 Go to: 1 1 - 10 of 1131

This report was generated on 3/8/21 at 8:18:58 AM - Refresh Report

Figure 12. Mobile device use for all users accessing No Money No Time from 17 July 2019 to 17 July 2020.

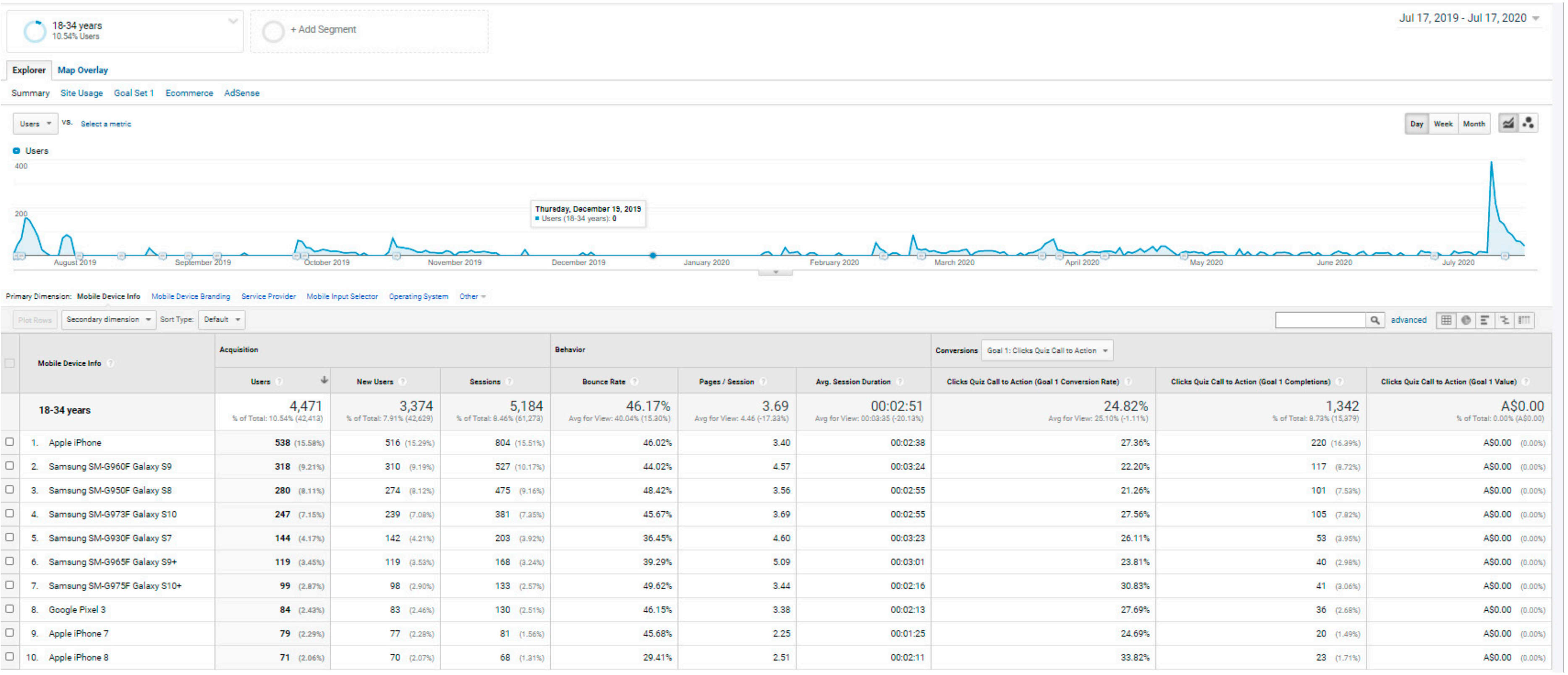

Mobile Device Info	Acquisition			Behavior			Conversions		
	Users	New Users	Sessions	Bounce Rate	Pages / Session	Avg. Session Duration	Clicks Quiz Call to Action (Goal 1 Conversion Rate)	Clicks Quiz Call to Action (Goal 1 Completions)	Clicks Quiz Call to Action (Goal 1 Value)
**18-34 years**	4,471<br>% of Total: 10.54% (42,413)	3,374<br>% of Total: 7.91% (42,629)	5,184<br>% of Total: 8.46% (61,273)	46.17%<br>Avg for View: 40.04% (15.30%)	3.69<br>Avg for View: 4.46 (-17.33%)	00:02:51<br>Avg for View: 00:03:35 (-20.13%)	24.82%<br>Avg for View: 25.10% (-1.11%)	1,342<br>% of Total: 8.73% (15,379)	A$0.00<br>% of Total: 0.00% (A$0.00)
1. Apple iPhone	**538** (15.58%)	516 (15.29%)	804 (15.51%)	46.02%	3.40	00:02:38	27.36%	220 (16.39%)	A$0.00 (0.00%)
2. Samsung SM-G960F Galaxy S9	**318** (9.21%)	310 (9.19%)	527 (10.17%)	44.02%	4.57	00:03:24	22.20%	117 (8.72%)	A$0.00 (0.00%)
3. Samsung SM-G950F Galaxy S8	**280** (8.11%)	274 (8.12%)	475 (9.16%)	48.42%	3.56	00:02:55	21.26%	101 (7.53%)	A$0.00 (0.00%)
4. Samsung SM-G973F Galaxy S10	**247** (7.15%)	239 (7.08%)	381 (7.35%)	45.67%	3.69	00:02:55	27.56%	105 (7.82%)	A$0.00 (0.00%)
5. Samsung SM-G930F Galaxy S7	**144** (4.17%)	142 (4.21%)	203 (3.92%)	36.45%	4.60	00:03:23	26.11%	53 (3.95%)	A$0.00 (0.00%)
6. Samsung SM-G965F Galaxy S9+	**119** (3.45%)	119 (3.53%)	168 (3.24%)	39.29%	5.09	00:03:01	23.81%	40 (2.98%)	A$0.00 (0.00%)
7. Samsung SM-G975F Galaxy S10+	**99** (2.87%)	98 (2.90%)	133 (2.57%)	49.62%	3.44	00:02:16	30.83%	41 (3.06%)	A$0.00 (0.00%)
8. Google Pixel 3	**84** (2.43%)	83 (2.46%)	130 (2.51%)	46.15%	3.38	00:02:13	27.69%	36 (2.68%)	A$0.00 (0.00%)
9. Apple iPhone 7	**79** (2.29%)	77 (2.28%)	81 (1.56%)	45.68%	2.25	00:01:25	24.69%	20 (1.49%)	A$0.00 (0.00%)
10. Apple iPhone 8	**71** (2.06%)	70 (2.07%)	68 (1.31%)	29.41%	2.51	00:02:11	33.82%	23 (1.71%)	A$0.00 (0.00%)

Figure 13. Mobile device use for young adults (18–34 years) accessing No Money No Time from 17 July 2019 to 17 July 2020.

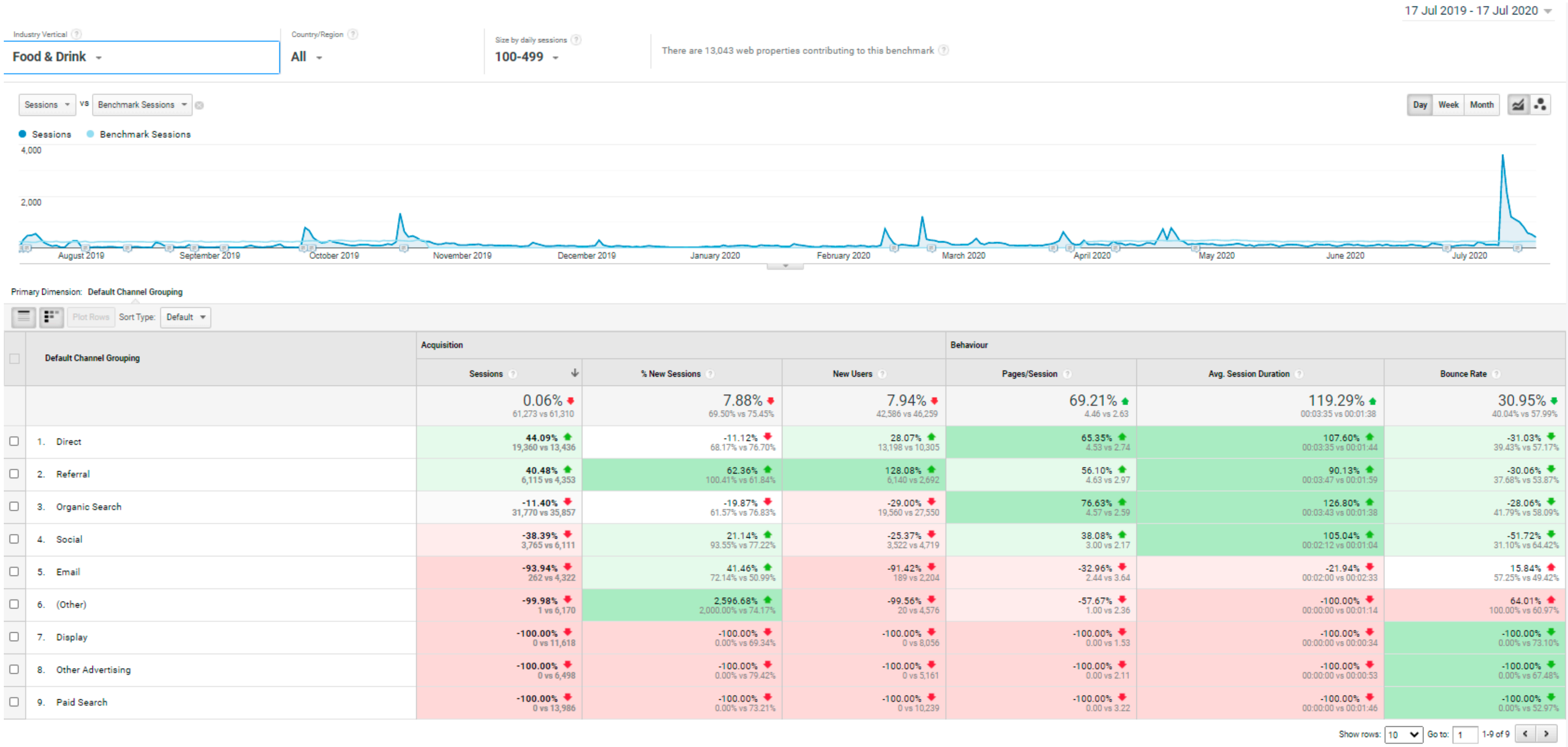

Default Channel Grouping	Acquisition: Sessions	Acquisition: % New Sessions	Acquisition: New Users	Behaviour: Pages/Session	Behaviour: Avg. Session Duration	Behaviour: Bounce Rate
	0.06% 61,273 vs 61,310	7.88% 69.50% vs 75.45%	7.94% 42,586 vs 46,259	69.21% 4.46 vs 2.63	119.29% 00:03:35 vs 00:01:38	30.95% 40.04% vs 57.99%
1. Direct	44.09% 19,360 vs 13,436	-11.12% 68.17% vs 76.70%	28.07% 13,198 vs 10,305	65.35% 4.53 vs 2.74	107.60% 00:03:35 vs 00:01:44	-31.03% 39.43% vs 57.17%
2. Referral	40.48% 6,115 vs 4,353	62.36% 100.41% vs 61.84%	128.08% 6,140 vs 2,692	56.10% 4.63 vs 2.97	90.13% 00:03:47 vs 00:01:59	-30.06% 37.68% vs 53.87%
3. Organic Search	-11.40% 31,770 vs 35,857	-19.87% 61.57% vs 76.83%	-29.00% 19,560 vs 27,550	76.63% 4.57 vs 2.59	126.80% 00:03:43 vs 00:01:38	-28.06% 41.79% vs 58.09%
4. Social	-38.39% 3,765 vs 6,111	21.14% 93.55% vs 77.22%	-25.37% 3,522 vs 4,719	38.08% 3.00 vs 2.17	105.04% 00:02:12 vs 00:01:04	-51.72% 31.10% vs 64.42%
5. Email	-93.94% 262 vs 4,322	41.46% 72.14% vs 50.99%	-91.42% 189 vs 2,204	-32.96% 2.44 vs 3.64	-21.94% 00:02:00 vs 00:02:33	15.84% 57.25% vs 49.42%
6. (Other)	-99.98% 1 vs 6,170	2,596.68% 2,000.00% vs 74.17%	-99.56% 20 vs 4,576	-57.67% 1.00 vs 2.36	-100.00% 00:00:00 vs 00:01:14	64.01% 100.00% vs 60.97%
7. Display	-100.00% 0 vs 11,618	-100.00% 0.00% vs 69.34%	-100.00% 0 vs 8,056	-100.00% 0.00 vs 1.53	-100.00% 00:00:00 vs 00:00:34	-100.00% 0.00% vs 73.10%
8. Other Advertising	-100.00% 0 vs 6,498	-100.00% 0.00% vs 79.42%	-100.00% 0 vs 5,161	-100.00% 0.00 vs 2.11	-100.00% 00:00:00 vs 00:00:53	-100.00% 0.00% vs 67.48%
9. Paid Search	-100.00% 0 vs 13,986	-100.00% 0.00% vs 73.21%	-100.00% 0 vs 10,239	-100.00% 0.00 vs 3.22	-100.00% 00:00:00 vs 00:01:46	-100.00% 0.00% vs 52.97%

Figure 14. Google analytics 'benchmarking' comparison of traffic acquisition for 'No Money No Time' with aggregated industry data from 13,034 food and drink websites from 17 July 2019 to 17 July 2020.

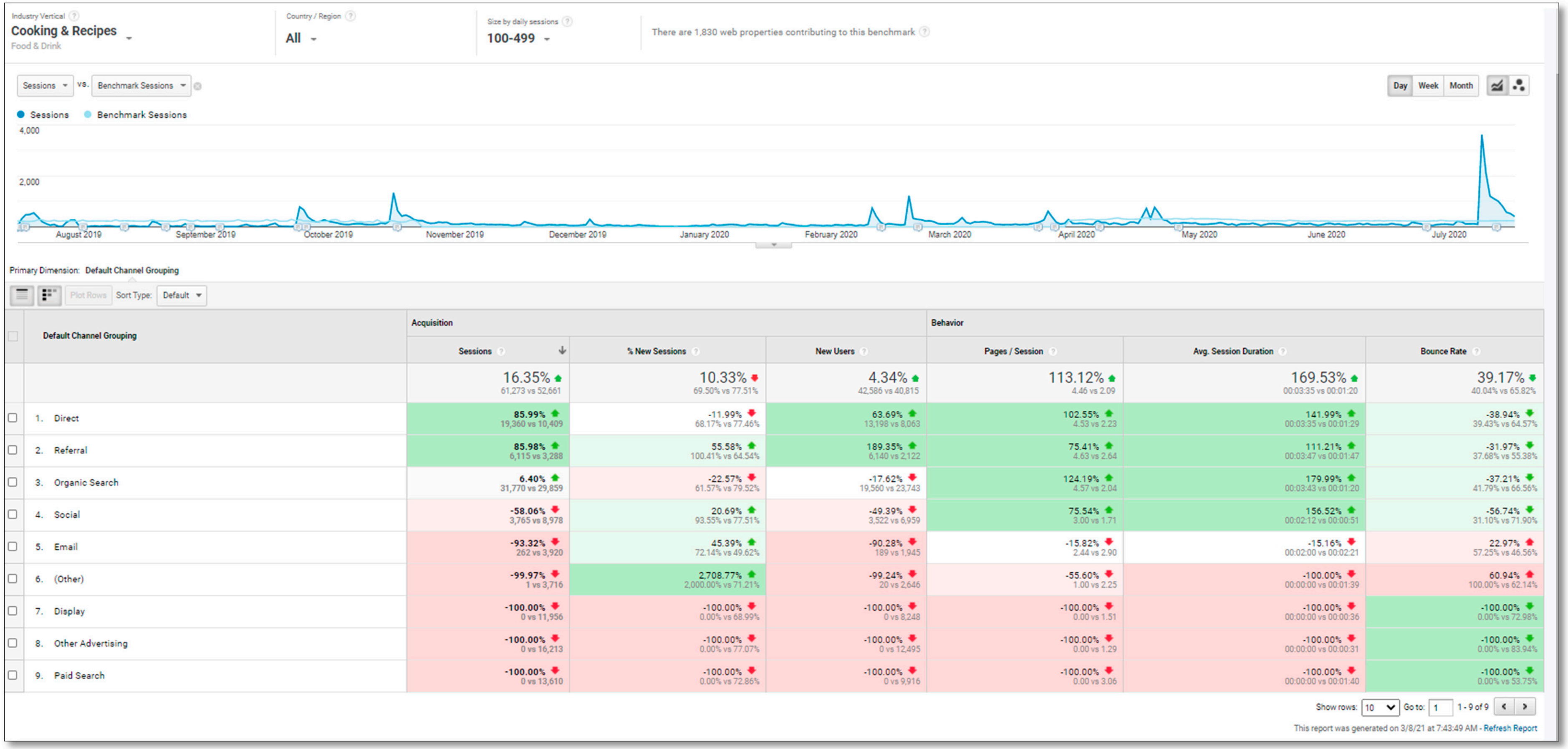

Default Channel Grouping	Acquisition			Behavior		
	Sessions	% New Sessions	New Users	Pages / Session	Avg. Session Duration	Bounce Rate
	16.35% 61,273 vs 52,661	10.33% 69.50% vs 77.51%	4.34% 42,586 vs 40,815	113.12% 4.46 vs 2.09	169.53% 00:03:35 vs 00:01:20	39.17% 40.04% vs 65.82%
1. Direct	85.99% 19,360 vs 10,409	-11.99% 68.17% vs 77.46%	63.69% 13,198 vs 8,063	102.55% 4.53 vs 2.23	141.99% 00:03:35 vs 00:01:29	-38.94% 39.43% vs 64.57%
2. Referral	85.98% 6,115 vs 3,288	55.58% 100.41% vs 64.54%	189.35% 6,140 vs 2,122	75.41% 4.63 vs 2.64	111.21% 00:03:47 vs 00:01:47	-31.97% 37.68% vs 55.38%
3. Organic Search	6.40% 31,770 vs 29,859	-22.57% 61.57% vs 79.52%	-17.62% 19,560 vs 23,743	124.19% 4.57 vs 2.04	179.99% 00:03:43 vs 00:01:20	-37.21% 41.79% vs 66.56%
4. Social	-58.06% 3,765 vs 8,978	20.69% 93.55% vs 77.51%	-49.39% 3,522 vs 6,959	75.54% 3.00 vs 1.71	156.52% 00:02:12 vs 00:00:51	-56.74% 31.10% vs 71.90%
5. Email	-93.32% 262 vs 3,920	45.39% 72.14% vs 49.62%	-90.28% 189 vs 1,945	-15.82% 2.44 vs 2.90	-15.16% 00:02:00 vs 00:02:21	22.97% 57.25% vs 46.56%
6. (Other)	-99.97% 1 vs 3,716	2,708.77% 2,000.00% vs 71.21%	-99.24% 20 vs 2,646	-55.60% 1.00 vs 2.25	-100.00% 00:00:00 vs 00:01:39	60.94% 100.00% vs 62.14%
7. Display	-100.00% 0 vs 11,956	-100.00% 0.00% vs 68.99%	-100.00% 0 vs 8,248	-100.00% 0.00 vs 1.51	-100.00% 00:00:00 vs 00:00:36	-100.00% 0.00% vs 72.98%
8. Other Advertising	-100.00% 0 vs 16,213	-100.00% 0.00% vs 77.07%	-100.00% 0 vs 12,495	-100.00% 0.00 vs 1.29	-100.00% 00:00:00 vs 00:00:31	-100.00% 0.00% vs 83.94%
9. Paid Search	-100.00% 0 vs 13,610	-100.00% 0.00% vs 72.86%	-100.00% 0 vs 9,916	-100.00% 0.00 vs 3.06	-100.00% 00:00:00 vs 00:01:40	-100.00% 0.00% vs 53.75%

Figure 15. Google analytics 'benchmarking' comparison of traffic acquisition 'No Money No Time' with aggregated industry data from 1830 websites with 'Cooking & Recipes' category from 17 July 2019 to 17 July 2020.

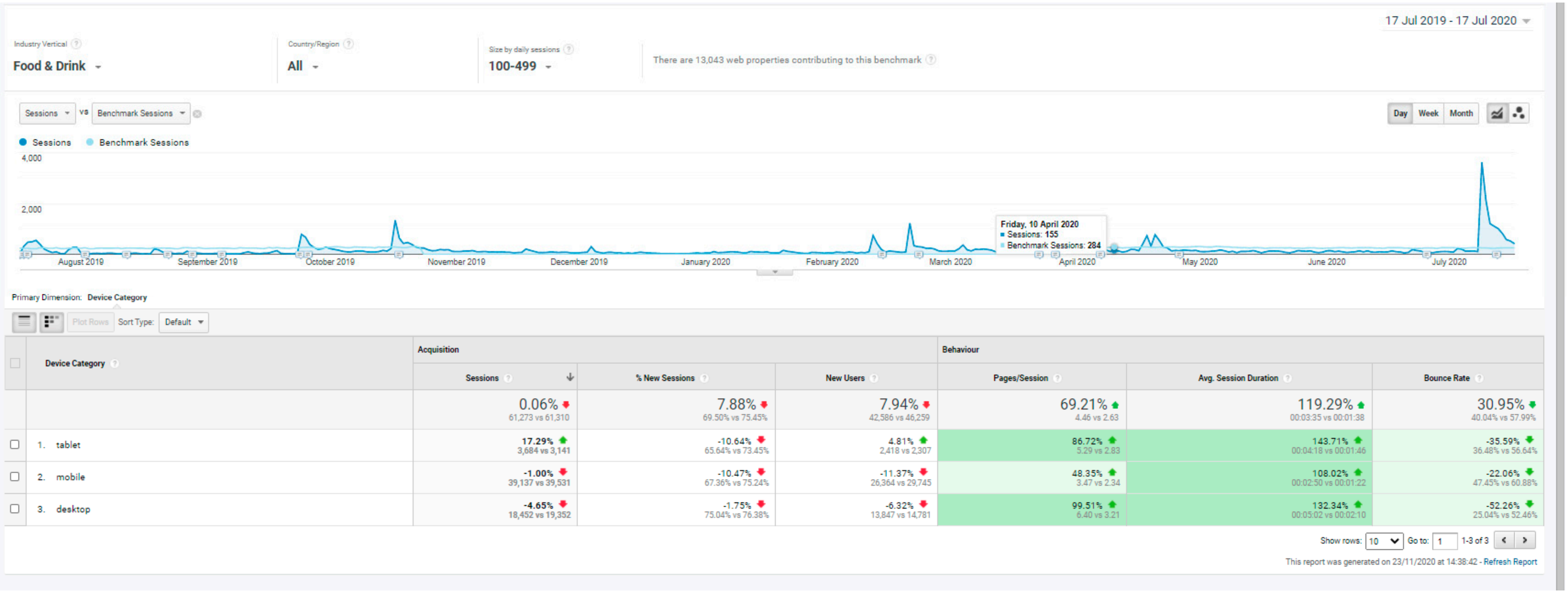

Figure 16. Google analytics 'benchmarking' comparison of device type recruitment and engagement data of 'No Money No Time' with aggregated industry data from 13,034 food and drink websites from 17 July 2019 to 17 July 2020.
